# Serum Kynurenine Pathway Metabolites as Candidate Diagnostic Biomarkers for Pituitary Adenoma: A Case–Control Study

**DOI:** 10.3390/medicina61122120

**Published:** 2025-11-28

**Authors:** Nur Düzen Oflas, Halil İbrahim Akbay, Murat Alay, Mehmet Erdem

**Affiliations:** 1 Department of Internal Medicine, Faculty of Medicine, University of Van Yuzuncu Yil, 65090 Van, Türkiye; dr.mehmet_erdem@hotmail.com; 2 Department of Biochemistry, Faculty of Medicine, University of Van Yuzuncu Yil, 65090 Van, Türkiye; 3 Department of Endocrinology, Faculty of Medicine, University of Van Yuzuncu Yil, 65090 Van, Türkiye

**Keywords:** pituitary adenoma, kynurenine pathway, diagnosis, predictive value

## Abstract

*Background and Objectives*: Pituitary adenomas are common intracranial tumors lacking specific non-invasive biomarkers. This study aimed to determine whether key metabolites and enzymes of the kynurenine pathway—including indoleamine 2,3-dioxygenase (IDO), kynurenine (KYN), kynurenic acid (KYNA), kynurenine aminotransferase (KAT), quinolinic acid, and picolinic acid—can serve as diagnostic biomarkers distinguishing patients with pituitary adenomas from healthy controls. *Materials and Methods*: We conducted a single-center, cross-sectional, case–control study with 50 patients with pituitary adenomas and 35 healthy controls. Serum levels of IDO, KYN, KYNA, KAT, quinolinic acid, and picolinic acid were measured via enzyme-linked immunosorbent assay (ELISA). Statistical analyses included group comparisons (*t*-test/Mann–Whitney U), multivariate logistic regression to identify independent predictors, receiver operating characteristic (ROC) curve analysis to evaluate diagnostic performance (area under the curve, AUC), and partial least squares discriminant analysis (PLS-DA) for multivariate metabolic profiling. *Results*: Serum kynurenine, kynurenic acid, 3-hydroxykynurenine, picolinic acid, IDO and kynureninase were significantly higher in the pituitary adenoma group than in healthy controls (*p* < 0.001), while tryptophan, kynurenine aminotransferase, anthranilic acid and quinolinic acid showed no significant differences. ROC analysis demonstrated excellent diagnostic accuracy, with KAT (AUC = 0.923) and KYNA (AUC = 0.901) showing the highest discrimination. Multivariate logistic regression identified IDO, KYN, and KYNA as independent predictors of pituitary adenoma (*p* < 0.05). PLS-DA of the combined metabolite data also demonstrated clear separation between patients and controls, confirming distinct metabolic profiles between the groups. *Conclusions*: Kynurenine pathway metabolites and enzymes show strong potential as non-invasive biomarkers for pituitary adenomas. In particular, elevated KAT and KYNA levels demonstrated high diagnostic performance. These findings suggest that a panel of kynurenine pathway metabolites could aid in the early, non-invasive detection of pituitary adenomas.

## 1. Introduction

Pituitary adenomas are one of the most common brain tumors, accounting for approximately 18% of all intracranial neoplasms [1,2]. Their prevalence is estimated at 115 cases per 100,000 in the general population, and although the vast majority are histologically benign, they can cause significant morbidity through neurological symptoms (e.g., visual field defects) due to mass effect and hormonal dysregulation, such as acromegaly and Cushing’s disease [1]. To better understand the clinical management and prognosis of these tumors, pituitary adenomas were redefined as pituitary neuroendocrine tumors (PitNETs) in the World Health Organization (WHO) 2022 classification, and clinicopathological scoring systems such as PANOMEN-3 were developed [3,4]. These advances highlight the need for new biomarkers that better reflect the biological behavior of pituitary adenomas and can guide treatment strategies.

The kynurenine pathway is the main catabolic route for more than 95% of dietary tryptophan [5]. It is initiated by the rate-limiting enzymes indoleamine-2,3-dioxygenase (IDO) and tryptophan-2,3-dioxygenase (TDO), which convert tryptophan to kynurenine via N-formylkynurenine [6]. Kynurenine occupies a central branching point, where it is further metabolized to kynurenic acid, anthranilic acid and 3-hydroxykynurenine through distinct enzymatic steps [7]. Downstream, quinolinic and picolinic acids are generated and contribute to de novo NAD^+^ synthesis.

Beyond its physiological functions, aberrant activation of the kynurenine pathway has been implicated in tumor progression and immune escape in several malignancies [8]. Increased IDO expression and accumulation of kynurenine can deplete tryptophan, impair effector T and NK cell responses, favor regulatory immune populations and activate Aryl Hydrocarbon Receptor (AHR)-dependent oncogenic signaling, thereby promoting cancer cell survival and proliferation [9,10].

Given that pituitary adenomas are proliferative neoplasms and the established role of the kynurenine pathway in promoting proliferation and immune tolerance in cancer, we hypothesized that this pathway may also play a role in the pathophysiology of pituitary adenomas. Therefore, the aim of this study was to comprehensively compare serum levels of key kynurenine pathway metabolites and enzymes between patients with pituitary adenomas and healthy controls and to investigate the value of these molecules as potential new, non-invasive diagnostic biomarkers for the disease.

## 2. Materials and Methods

### 2.1. Study Population

This single-center, prospective, observational, cross-sectional case–control diagnostic accuracy study was conducted at Van Yüzüncü Yıl University. Patients with pituitary adenoma and healthy controls were consecutively enrolled, and a single venous blood sample was obtained preoperatively from each patient (before any surgery or radiotherapy) and at a single visit from each control. The study protocol was approved by the Van Yüzüncü Yıl University Clinical Research Local Ethics Committee (date: 20 December 2024, decision number: 2024/13-03), and written informed consent was obtained from all participants.

The diagnosis of pituitary adenoma was made by evaluating clinical/biochemical findings and imaging results together. Pituitary MRI (sellar/parasellar, thin-section, gadolinium-enhanced, pituitary protocol) was performed, and lesions were classified as microadenoma (<10 mm) or macroadenoma (≥10 mm). Basal hormone profiles and, if necessary, dynamic tests were performed based on clinical suspicion: growth hormone suppression and IGF-1 in the oral glucose tolerance test for acromegaly; 1 mg (overnight dexamethasone) ODN or late-night salivary cortisol/24 h urinary free cortisol for Cushing’s disease; serum prolactin (excluding macroprolactin) for prolactinoma; and cases without hypersecretion were considered nonfunctional. Computerized visual field testing was performed for visual complaints or suspicion of chiasmal compression. Secondary causes of hyperprolactinemia (hypothyroidism, etc.) and systemic comorbidities were clinically excluded.

The healthy control (HC) group was selected from volunteers within the institution, ensuring a similar distribution to the patients in terms of age and gender. Individuals were included without any endocrine disease, active infection, chronic inflammatory disease, malignancy, renal/liver failure, or regular steroid use.

### 2.2. Inclusion and Exclusion Criteria

Inclusion: (i) age 18–70 years, (ii) detection of lesions by MRI in the PA group and endocrine evaluation consistent with the above international diagnostic guidelines, and (iii) availability of serum samples.

Exclusions: (i) pregnancy or lactation, (ii) active infection/autoimmune disease, (iii) severe renal/liver failure, (iv) systemic steroid/chemo-radiotherapy in the last 3 months, (v) chronic psychiatric/neurological diseases or use of medications affecting tryptophan/nicotinamide metabolism, and (vi) cases lacking data/sample integrity.

### 2.3. Measurement of Tryptophan and Kynurenine Pathways Metabolites and Related Enzymes

At the time of hospital admission (preoperatively) and approximately 1 month after surgery (after an overnight fast), 5 mL of venous blood was collected from each participant from the antecubital vein into dry tubes/serum separator tubes (SSTs) according to clinical protocol. The samples were quickly transferred to the laboratory and were coagulated at room temperature for 30 min and then centrifuged at 2500× *g* for 10 min. Then serum was transferred to polypropylene tubes and stored at −80 °C for nearly uniform durations for all samples until analysis.

The upper serum phase was transferred to polypropylene tubes and stored at −80 °C until analysis. Only preoperative samples were used in the analyses.

Serum tryptophan, kynurenine, kynurenic acid (KYNA), picolinic acid, quinolinic acid, indoleamine-2,3-dioxygenase (IDO), kynureninase, kynurenine aminotransferase (KAT), and 3-hydroxykynurenineninase levels were measured using Reed Biotech (Hubei, Wuhan, China) ELISA kits according to the manufacturer’s instructions. Absorbances were read at 450 nm using a BioTek Elx-800 (Winooski, VT, USA) microplate reader; concentrations were calculated from standard curves generated for each analyte.

Selected analytical characteristics according to manufacturer’s claims: Tryptophan sensitivity 0.62 µg/mL, measuring range 1.57–100 µg/mL; Kynurenine 0.94 µg/mL, 1.56–200 µg/mL; KYNA 9.24 ng/mL, 31.25–2000 ng/mL; Picolinic acid 35.16 ng/mL, 78.13–5000 µmol/mL; Quinolinic acid 13.7 mmol/mL, 31.25–2000 mmol/mL; IDO 0.118 ng/mL, 0.32–20 ng/mL. (According to the manufacturers’ datasheets, the repeatability of these ELISAs is reported as a coefficient of variation <10%. Measurement performances of other kits are in accordance with the manufacturer’s documentation.)

### 2.4. Statistical Analysis

Statistical analyses were performed using IBM SPSS Statistics v23.0 (IBM Corp., Armonk, NY, USA), Analyse-it (Microsoft Excel add-in), MedCalc v23.3.7 (64-bit), and MetaboAnalyst (www.metaboanalyst.ca). Distributions of continuous variables were assessed using the Shapiro–Wilk test and visual inspection (histograms and Q–Q plots); normally distributed variables were summarized as mean ± standard deviation, and non-normally distributed variables as median (interquartile range). In comparisons of two independent groups, the Independent Samples *t*-test (with Welch correction applied when necessary) was used for normally distributed data, and the Mann–Whitney U test was used for non-normally distributed data. Categorical variables were compared using the chi-square test or Fisher’s exact test when appropriate. Relationships between tumor diameter and kynurenine pathway metabolites and enzymes were examined using the Pearson correlation coefficient and reported with 95% confidence intervals.

Binary logistic regression was performed to identify predictors of pituitary adenoma presence (patient = 1, healthy = 0). In univariate models, each biomarker was evaluated separately, and the crude odds ratio (OR) and 95% confidence interval (CI) were calculated. In the multivariate model, age and sex were required a priori covariates; variables of clinical significance and/or considered candidates in univariate screening were included together. Multicollinearity was assessed using tolerance and variance inflation factor (VIF); only one variable representing the same biological pathway and exhibiting collinearity was included in the model. Given the limited number of events (50 pituitary adenoma cases), to reduce the risk of overfitting, we prespecified a parsimonious multivariable model including sex, age, and up to three biomarker predictors. Candidate metabolites were first screened in univariable logistic regression; those with *p* < 0.10 and with strong biological plausibility in the kynurenine pathway (IDO, kynurenic acid, and kynurenine) were retained in the final model, ensuring an events-per-variable ratio of approximately 10. Model fit was assessed using the Hosmer–Lemeshow test, and discrimination was assessed using the area under the ROC curve (AUC); coefficients were reported as adjusted OR (95% CI). Partial Least Squares Discriminant Analysis (PLS-DA), a supervised multivariate method, was applied to examine the metabolic distinction between the groups. Prior to analysis, all continuous variables were centered and scaled to unit variance (autoscale). The number of components was determined by cross-validation; model validity was tested with permutation testing and CV-ANOVA. The contribution of variables to the model was assessed using Variable Importance in Projection (VIP) scores, and a VIP score ≥ 1 was considered significant.

The diagnostic performance of the biomarkers was investigated using ROC analysis. AUC values (95% CI) were calculated using the DeLong method. The optimal threshold values were determined using the Youden index; sensitivity and specificity at these thresholds were reported with 95% confidence intervals (Clopper–Pearson). To minimize optimistic bias in the ROC analysis, we additionally calculated a ten-fold cross-validated ROC curve and AUC for the final multivariable logistic regression model (sex, age, IDO, kynurenic acid, and kynurenine). In each fold, the model was fitted to 90% of the data, and predicted probabilities were obtained for the held-out 10%; the pooled cross-validated probabilities were then used to construct the ROC curve and estimate the cross-validated AUC (pROC package in R).

All tests were two-sided, and statistical significance was set at *p* < 0.05.

## 3. Results

The clinical and demographic data for the patient group and comparison of tryptophan and kynurenine pathway metabolites with those of healthy controls are presented in Table 1.

**Figure 1:**
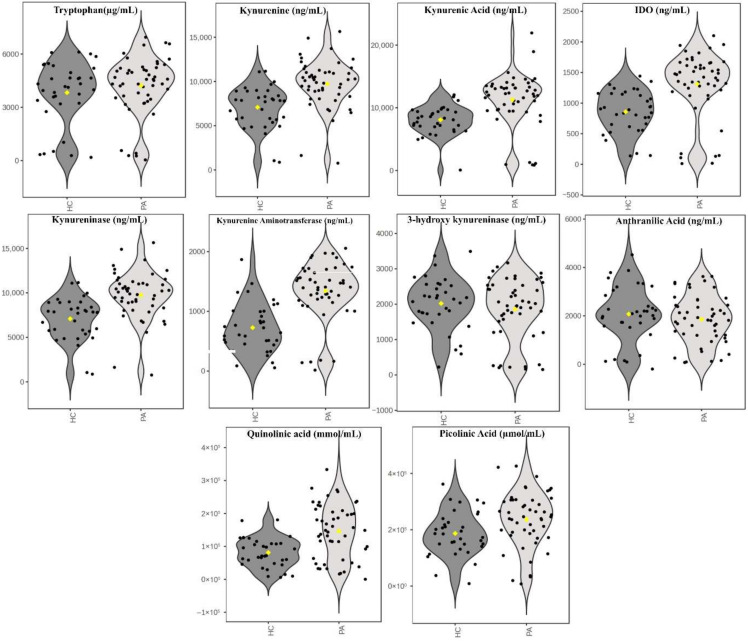
Distributions of major metabolites and enzyme levels of the kynurenine pathway in the HC and PA groups (violin plots). HC: Healthy Control Group; PA: Pituitary Adenoma Patient Group.

No statistically significant differences were found between the pituitary adenoma patient group and the healthy control group in terms of age and gender. When serum levels of tryptophan and kynurenine pathway metabolites were examined, no statistically significant differences were found between the groups in terms of tryptophan, kynureninease, 3-hydroxykynurenine, and anthranilic acid levels. Kynurenine, kynurenic acid, IDO, kynurenine aminotransferase, quinolinic acid, and picolinic acid levels were statistically significantly higher in the pituitary adenoma patient group than in the healthy control group (Figure 1).

**Figure 2:**
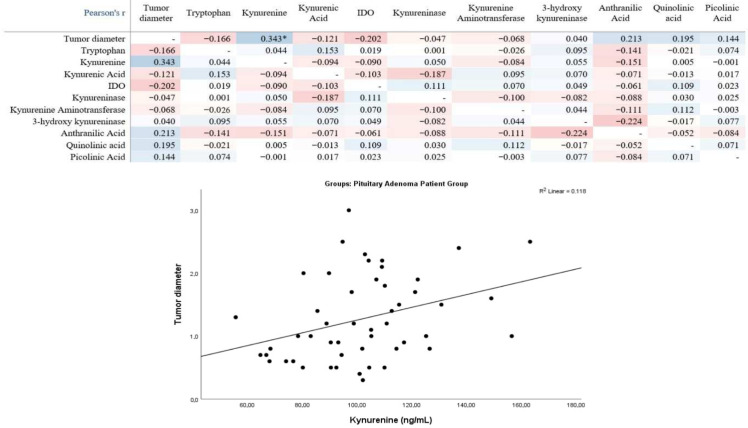
Correlation between tumor diameter and kynurenine pathway metabolite and enzyme levels in pituitary adenoma patients: Pearson correlation heat map and scatter plot between kynurenine and tumor diameter. * *p* < 0.05 are statistically significant.

Pearson correlation analysis in the pituitary adenoma patient group revealed a statistically significant positive correlation between tumor diameter and kynurenine levels. No statistically significant correlations were observed between tumor diameter and tryptophan, kynurenic acid, indoleamine-2,3-dioxygenase (IDO), kynureninase, kynurenine aminotransferase, 3-hydroxykynureninase, anthranilic acid, quinolinic acid, or picolinic acid. Other pairwise correlations between metabolites and enzymes were mostly weak and not statistically significant. The linear trend in the scatterplot was consistent with the direction of the positive correlation between tumor diameter and kynurenine (Figure 2).

Univariate and multivariate binary logistic regression analyses were performed to identify potential biomarkers associated with the presence of a pituitary adenoma (patient group = 1, healthy control group = 0). According to the univariate analysis results, a statistically significant association was found between the presence of a pituitary adenoma and six biomarkers: increased quinolinic acid (mmol/mL) (odds ratio [OR] = 1.002, 95% confidence interval [CI]: 1.001–1.003, *p* = 0.001), picolinic acid (µmol/mL) (OR = 1.001, 95% CI: 1.000–1.002, *p* = 0.004), indoleamine 2,3-dioxygenase (IDO) (ng/mL) (OR = 1.688, 95% CI: 1.349–2.111, *p* = 0.004), kynurenic acid (ng/mL) (OR = 1.091, 95% CI: 1.053–1.132, *p* = 0.001), kynurenine (ng/mL) (OR = 1.065, 95% CI: 1.034–1.097, *p* = 0.001) and kynurenine aminotransferase (ng/mL) (OR = 1.813, 95% CI: 1.413–2.319, *p* < 0.001).

Gender, age, tryptophan, kynurenine, 3-hydroxykynurenine, and anthranilic acid levels were not found to be statistically significantly associated with the presence of pituitary adenoma (*p* > 0.05 for all). All univariate analysis results are summarized in the table above.

Variables found to be significant in the univariate analysis and considered clinically important were included in the multivariate logistic regression model to identify independent predictors of pituitary adenoma.

According to the model results, IDO (ng/mL) (OR = 2.736, 95% CI: 1.223–6.122, *p* = 0.014), kynurenic acid (ng/mL) (OR = 1.155, 95% CI: 1.031–1.293, *p* = 0.013), and kynurenine (ng/mL) (OR = 1.104, 95% CI: 0.992–1.229, *p* = 0.009) levels were determined to be statistically significant and independent predictors of pituitary adenoma. This finding shows that, in this case–control setting, each one-unit increase in IDO, kynurenic acid, and kynurenine levels is associated with 2.74-fold, 1.16-fold, and 1.10-fold higher odds of being in the pituitary adenoma group rather than the control group, respectively. Quinolinic acid and picolinic acid, which were found to be significant in the univariate analysis, lost statistical significance when included in the multivariate model (*p* = 0.128 and *p* = 0.754, respectively). Detailed results of the multivariate analysis are presented in the table below (Table 2).

Partial Least Squares Discriminant Analysis (PLS-DA), a supervised multivariate statistical method, was applied to determine the overall differences in kynurenine pathway metabolite profiles between patients with pituitary adenomas and healthy controls and to identify the metabolites most effective in distinguishing between the groups. PLS-DA models with 1–5 components were evaluated by 10-fold cross-validation. A 5-component model was selected because it provided slightly higher classification accuracy while maintaining stable Q^2^ values, and additional components beyond 5 did not meaningfully improve model performance (Supplementary Table S1). The final 5-component PLS-DA model achieved a cross-validated accuracy of 0.78472 (78%), with R^2^ = 0.54 and Q^2^ = 0.0.22.

**Figure 3:**
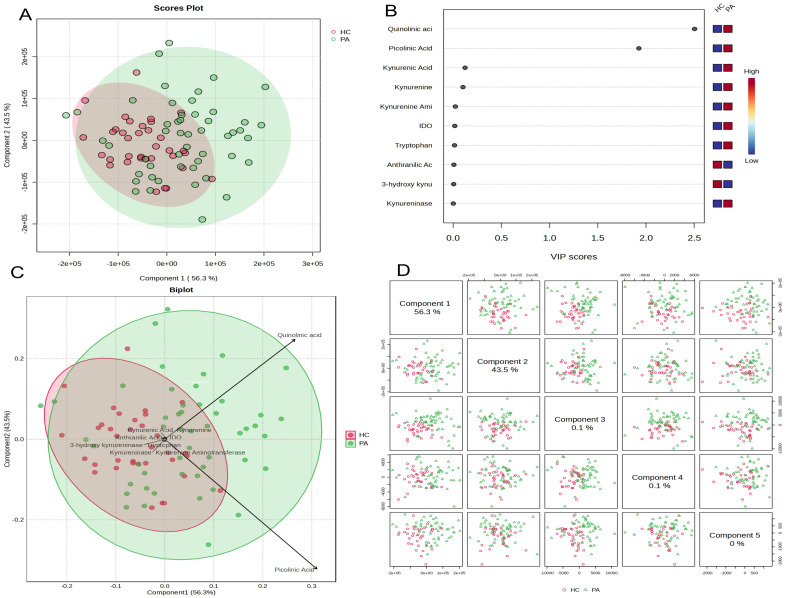
Partial Least Squares Discriminant Analysis (PLS-DA) Based on Kynurenine Pathway Metabolite Profiles in Pituitary Adenoma Patients (PA) and Healthy Controls (HCs). (**A**) Scores Plot showing the separation of groups according to the first two components of the model (Component 1 and Component 2). Ellipses represent 95% confidence intervals. (**B**) Variable Importance in Projection (VIP) scores plot ranking the importance of metabolites in group separation. Color boxes indicate the relative concentration (high/low) of the respective metabolite between groups. (**C**) Biplot analysis showing the relationship between observations (patients and controls) and variables (metabolites) simultaneously. Vectors indicate the direction and effect of each metabolite in the model. (**D**) Matrix containing score plots of the first five components of the model.

The PLS-DA model’s scores plot showed a significant tendency for differentiation between the two groups (Figure 3A). The first two components of the model (Component 1 and Component 2) explained 56.3% and 43.5% of the total variance in the data, respectively. Although the PA patient group (red circles) and the HC group (green circles) tended to cluster in different quadrants, a partial transition region was also observed between the two groups. This suggests a clear metabolic distinction between the groups and also indicates the presence of individual variation.

Variable Importance in Projection (VIP) scores were calculated to identify the metabolites that played the most significant role in distinguishing between the groups. Variables with VIP scores greater than 1.0 were considered to provide the most statistically significant contribution to the distinction between groups. The analysis revealed that quinolinic acid (VIP = 2.8), picolinic acid (VIP = 2.0), and kynurenic acid (VIP = 1.8) stood out as the metabolites with the highest VIP scores (Figure 3B). These variables were followed by kynurenine, kynurenine aminotransferase, and IDO, respectively. The color scale indicates that quinolinic acid and picolinic acid levels were higher in the PA group compared to the HC group (red), while other significant metabolites were relatively higher in the HC group (blue).

Biplot analysis combining score and loading plots visually demonstrated which metabolites influenced the group distinction in which direction (Figure 3C). The extension of the quinolinic acid and picolinic acid vectors toward the region where the PA patient group clusters confirms that these two metabolites are positively correlated with the PA group and that their increases in this group play a key role in discrimination. On the other hand, metabolites such as 3-hydroxykynurenine, kynurenine, and kynurenine aminotransferase are located closer to the region where the HC group clusters.

Finally, when the score plots for the first five components in the model are examined, it is seen that the clearest distinction between the groups is provided by the first two components, while the variance explained by the subsequent components (Components 3, 4, and 5) is very low (0.1%, 0.1%, and 0%) and does not contribute significantly to distinguishing the groups (Figure 3D). This confirms that the first two components of the PLS-DA model successfully summarize the metabolic differences between the groups.

**Figure 4:**
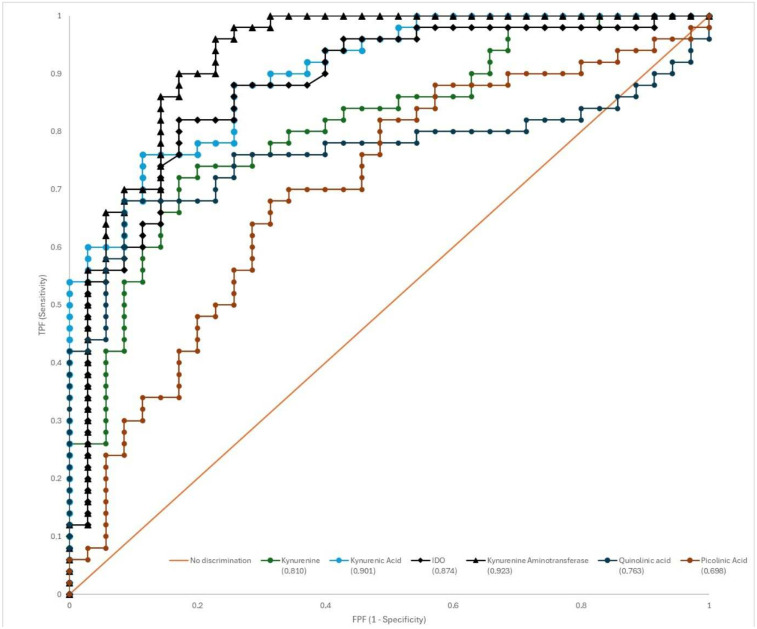
ROC Curve Analysis of Diagnostic Performance of Kynurenine Pathway Metabolites in Diagnosing Pituitary Adenoma.

We first conducted ROC analysis for tryptophan, kynurenine pathway metabolites, and related enzymes. ROC curves and diagnostic properties of variables with AUC values above 0.7 are presented in Table 3 and Figure 4. ROC curves for tryptophan (AUC = 0.56), kynureninase (AUC = 0.584), 3-hydroxykynureninase (AUC = 0.548), and anthranilic acid (AUC = 0.548), with AUC values below 0.7, are presented in Figure S1 (Supplementary Materials).

Receiver Operating Characteristic (ROC) curve analysis was performed to evaluate the potential of kynurenine pathway metabolites, which were found to be significant in univariate and multivariate analyses, to distinguish pituitary adenoma from healthy individuals. The diagnostic performance of six different metabolites is shown comparatively in Figure 4, and detailed numerical data from this analysis are summarized in Table 3. According to the ROC analysis results, kynurenine aminotransferase was determined to be the biomarker with the highest discrimination in the diagnosis of pituitary adenoma. Kynurenine aminotransferase exhibited a near-perfect diagnostic performance with an Area Under the Curve (AUC) value of 0.923 (95% Confidence Interval [CI]: 0.845–0.970). This was followed by kynurenic acid (AUC = 0.901, 95% CI: 0.816–0.955), which also demonstrated excellent diagnostic value, and IDO (AUC = 0.874, 95% CI: 0.785–0.936), with kynurenine (AUC = 0.810, 95% CI: 0.710–0.887) demonstrating good diagnostic performance. Quinolinic acid (AUC = 0.763) and picolinic acid (AUC = 0.698) showed lower diagnostic accuracy (Figure 4). The optimal cutoff, sensitivity, and specificity values for each metabolite in diagnosing pituitary adenoma are detailed in Table 3. The optimal cutoff value for kynurenine aminotransferase, which had the highest AUC value, was determined to be >10.08. At this value, a very high sensitivity of 96% (95% CI: 86–99) and specificity of 77% (95% CI: 60–89) were achieved. Similarly, when a threshold value of >100.59 was used for kynurenic acid, a sensitivity of 76% and a high specificity of 89% were obtained. These findings strongly support the notion that kynurenine aminotransferase and kynurenic acid, in particular, may be potential biomarkers for pituitary adenoma. The multivariable model showed excellent discrimination between pituitary adenoma and control subjects. The ten-fold cross-validated ROC curve yielded an AUC of 0.963 with a 95% confidence interval of 0.930–0.997 (Supplementary Figure S2), indicating that the high discriminative performance persisted after correction for overfitting.

## 4. Discussion

This study provides the first comprehensive evidence demonstrating that the kynurenine pathway of tryptophan metabolism is significantly activated in patients with pituitary adenomas (currently known as PitNETs). This finding is preliminary because, while the role of this critical immunometabolic pathway in malignant cancers has been extensively investigated, PitNETs remain largely unexplored. The key findings of our study revealed statistically significant increases in serum levels of the rate-limiting enzyme of the pathway, IDO, the central metabolite kynurenine, and the downstream metabolites kynurenic acid, quinolinic acid, and picolinic acid in the patient group compared to healthy controls.

The increase in IDO and kynurenine observed in our findings is consistent with a large body of literature demonstrating that this axis is the cornerstone of tumor immune escape [11]. Increased IDO activity leads to local tryptophan depletion, which is essential for T cell proliferation, leading to T cell anergy and cell cycle arrest [12]. Accumulated kynurenine promotes the generation of regulatory T cells (Treg) and suppresses effector T and NK cells [13]. Importantly, kynurenine is now recognized as an endogenous ligand of the Aryl Hydrocarbon Receptor (AHR), a transcription factor implicated in carcinogenesis, suggesting that this metabolic change constitutes a direct oncogenic signaling mechanism [14]. Kynurenine enters the cell, binds to the AHR, triggers its translocation to the nucleus, and initiates a transcriptional program that promotes cell proliferation, migration, and inhibits apoptosis [15]. There are no studies in the literature directly examining the serum levels of tryptophan and kynurenine pathway metabolites in pituitary adenomas, but they have been studied in different cancer types. Suzuki et al., in a study of 123 lung cancer patients and 45 healthy individuals, reported that tryptophan levels were significantly lower in cancer patients than in healthy individuals, while kynurenine levels were significantly higher. To demonstrate IDO activity, they calculated the kynurenine/tryptophan ratio and noted increased IDO activity in lung cancer patients [16]. Icer et al. measured only kynurenine levels in patients with benign and malignant prostate cancer and in healthy individuals and reported increased kynurenine levels in patients with malignant prostate cancer [17]. In our study, we also reported elevated kynurenine and IDO levels in patients with pituitary adenomas, and these results support the literature.

In our study, we observed a significant increase in kynurenic acid levels in patients with pituitary adenomas. Consistent with the reviewer’s point, kynurenic acid exhibits context-dependent biological roles: it may act as an oncometabolite through AHR activation and immune modulation, yet it also demonstrates anti-proliferative effects in certain tumor models by inhibiting ERK1/2, p38 MAPK, and AKT pathways. We have therefore revised the discussion to present a more balanced interpretation reflecting these dual actions [18]. Several studies have examined kynurenic acid levels in different cancers, showing variations between cancerous healthy tissues. Kynurenic acid is found in colon adenocarcinoma, glioblastoma, renal cell carcinoma, and oral squamous cell carcinoma [19,20,21].

Similarly, our interpretations of quinolinic acid and picolinic acid have been moderated. Elevated quinolinic acid may reflect systemic immune activation, NAD^+^ metabolic demand, or host–tumor metabolic interactions; however, serum levels alone cannot determine tumor-intrinsic metabolic reprogramming. Likewise, although animal studies report antitumor effects of picolinic acid, current human data remain limited, and thus its role in pituitary adenomas should be considered preliminary.

Kynureninase is a pyridoxal phosphate (PLP)-dependent hydrolase that catalyzes the hydrolytic cleavage of kynurenine and 3-hydroxykynurenine, yielding anthranilic acid and 3-hydroxyanthranilic acid, respectively. In our study, we found that the levels of kynureninease, 3-hydroxykynurenineninase, and anthranilic acid, which are all involved in this branch of the kynurenine pathway, were not statistically significantly different in patients with pituitary adenomas than in healthy individuals. However, kynurenine aminotransferase activity was found to be higher in patients with pituitary adenomas. This suggests a targeted enzymatic dysregulation rather than a general, nonspecific activation of the pathway. Specifically, the increase in kynurenine aminotransferase, which catalyzes the conversion of kynurenine to kynurenic acid, suggests that kynurenine is preferentially directed toward kynurenine aminotransferase–mediated kynurenic acid formation rather than toward other downstream branches of the pathway. In our study, the pattern of elevated kynurenine pathway metabolites in patients with pituitary adenoma is consistent with an upregulated systemic kynurenine pathway and a more immunoregulatory milieu; however, given the cross-sectional design and reliance on peripheral serum measurements, we cannot attribute these alterations directly to tumor-intrinsic metabolic reprogramming or infer causality.

Quinolinic acid, one of the end products of the kynurenine pathway, is an important metabolite involved in NAD^+^ synthesis. Our study found significantly increased serum quinolinic acid levels in patients with pituitary adenoma. This finding is consistent with a recent large prospective cohort study, which reported that high quinolinic acid levels in prediagnostic blood samples were significantly associated with the risk of developing lung cancer [22]. The researchers noted that quinolinic acid correlates with IDO activity in immune cells, and circulating QA levels are positively correlated with inflammatory markers (neopterin and the Kyn/Trp ratio). It has also been suggested that QA may inhibit T and natural killer (NK) cell proliferation, leading to immune suppression and thereby promoting tumor growth. This mechanism suggests that the high quinolinic acid levels detected in our study may contribute to the immune evasion potential of pituitary adenomas. Quolinic acid is also a precursor of NAD^+^ biosynthesis; it has been suggested that tumor cells may utilize this metabolite to meet their increased redox needs. Thus, QA may facilitate immune suppression while also serving as a “metabolic fuel” that supports the energy metabolism of tumor cells. This dual effect suggests that the increased quinolinic acid levels seen in pituitary adenomas may be associated with both metabolic reprogramming and immune tolerance.

A recent review compiling the “dual-faceted” role of the kynurenine pathway in cancer biology highlights the antitumor potential of picolinic acid: It has been reported that picolinic acid significantly reduces tumor growth and prolongs survival in a mouse model of Ehrlich ascites carcinoma and similarly provides a therapeutic effect in the MBL-2 lymphoma model. However, the mechanism of these effects is not clear, and the amount of modern data is limited. The presence of antitumor activity of picolinic acid has been demonstrated in an experimental animal study by Leuthauser et al. [23]. The antiproliferative activity of picolinic acid has been reported by Ruffmann et al. [24]. Considering the antitumor effect reported in animal experiments together with the high circulating picolinic acid levels in our human sample (probably via the immune–metabolic axis), picolinic acid may have value as a secondary biomarker in pituitary adenoma biology. This suggests that it may be an indicator of processes such as NAD^+^ demand/reprogrammed tryptophan catabolism reflected in the circulation.

Our study also evaluated the diagnostic performance of tryptophan and kynurenine pathway metabolites. Pituitary adenoma diagnosis relies on costly imaging methods and complex hormonal tests, and a simple, reliable blood-based biomarker represents a significant clinical need. Our study strongly demonstrates the potential of kynurenine pathway metabolites to fill this gap. Our ROC analysis results demonstrated the high diagnostic performance of two molecules in particular. Kynurenine aminotransferase exhibited an almost perfect diagnostic discriminatory power with an AUC of 0.923, demonstrating 96% sensitivity and 77% specificity at a cutoff point of >10.08 ng/mL. Similarly, kynurenic acid demonstrated a high diagnostic performance with an AUC of 0.901. Multivariate logistic regression analysis further strengthened these findings by confirming that IDO, kynurenine acid, and kynurenine were independent predictors of the presence of pituitary adenoma, independent of age and gender. The value of these biomarkers goes beyond simply indicating the presence of disease. Each marker in the panel reflects a different biological process: IDO represents immune activation/escape; kynurenine represents the production of an oncometabolite; and kynurenine aminotransferase/kynurenic acid represents a neuroprotective response or a specific metabolic shunt. PLS-DA analysis visually confirmed that a panel of these metabolites, rather than a single marker, effectively distinguishes patients from controls. Therefore, a kynurenine pathway biomarker panel may have the potential not only to establish a diagnosis but also to provide a non-invasive “liquid biopsy” of the tumor’s biological status. This approach may be valuable in the future for predicting tumor behavior or monitoring response to therapy.

Previous studies in different malignancies, including brain, lung, prostate and gastrointestinal tumors, have consistently shown that activation of the kynurenine pathway is associated with tumor progression, immune escape and adverse clinical outcomes [5,8,9,11,12,16,17]. Elevated circulating kynurenine levels and an increased kynurenine/tryptophan ratio have been reported in several solid tumors, such as lung and prostate cancer, and correlate with tumor burden and features of systemic immune suppression [16,17]. In addition, dysregulated levels of downstream metabolites, particularly kynurenic acid, have been demonstrated in tumor tissue or biological fluids from colon adenocarcinoma, glioblastoma, renal cell carcinoma and oral squamous cell carcinoma [19,20,21]. These observations are broadly consistent with our findings in pituitary adenomas and support the concept that dysregulation of the kynurenine pathway is a common feature of tumorigenesis. However, our data indicate that KAT activity and KYNA concentrations exhibit particularly high diagnostic performance in pituitary adenomas, underlining the importance of disease-specific evaluation when considering kynurenine pathway-related metabolites as potential non-invasive biomarkers.

While the results of this study provide important insights into the role of kynurenine pathway biomarkers in pituitary adenomas, there are some limitations that should be considered when interpreting the findings. The sample size was relatively small, and the study was conducted at a single center. This may limit the strength and generalizability of the statistical associations obtained. Similar analyses in larger, multicenter cohorts are necessary to confirm our findings and generalize them to the population. Second, the study used only preoperative serum samples, which are from a single time point. Changes in kynurenine pathway metabolite levels after surgical resection of the tumor or before and after medical treatment were not examined. Therefore, we cannot comment on the dynamic changes in these biomarkers. In the future, comparisons of preoperative and postoperative samples or monitoring of kynurenine pathway parameters during treatment response may illuminate the prognostic and predictive value of these biomarkers. Third, our study assessed systemic levels in serum and did not directly measure local kynurenine pathway enzyme expression within the tumor tissue. Although the serum Kyn/Trp ratio indirectly reflects IDO activity, examination of IDO or TDO expression in tumor tissue using immunohistochemical or molecular methods could provide important complementary data to support our findings. Finally, pituitary adenomas are a heterogeneous group; this study could not assess whether there are differences in kynurenine pathway activity between functional (hormone-secreting) and nonfunctional subtypes. Such a comparison was not possible due to the limited number of subgroups in our sample. Future studies examining kynurenine pathway metabolite profiles separately in hormone-active adenoma subtypes, such as acromegaly, Cushing’s disease, or prolactinoma, in larger series would be beneficial for the discovery of disease-specific biomarkers. Despite these limitations, our study is one of the pioneering studies addressing kynurenine pathway components in pituitary adenomas, and our data are biologically consistent with other tumor studies in the literature.

This study demonstrates for the first time that the kynurenine pathway of tryptophan metabolism is profoundly activated in patients with pituitary neuroendocrine tumors. This metabolic reprogramming, focusing on the IDO–kynurenine–AHR axis, offers a plausible mechanism for tumor growth and immune evasion, linking these “benign” neoplasms to the established hallmarks of malignancy. Most importantly, specific serum kynurenine pathway components, particularly kynurenine aminotransferase and kynurenic acid, are emerging as exceptionally powerful and promising non-invasive biomarkers for the diagnosis of pituitary adenomas. In conclusion, our study demonstrates that kynurenine pathway enzymes and metabolites may be a valuable new tool in the diagnosis of pituitary adenomas and provides important insights into the intersection of tumor immunology and metabolism. Accordingly, the mechanistic links discussed here should be regarded as hypothesis-generating, informed by prior experimental work, rather than causal relationships demonstrated by the present dataset, and they require confirmation in dedicated tissue-based work. Future validation of these components in larger cohorts and their potential for clinical use are crucial.

Limitations of the study: This study has several limitations. First, the control group consisted of institutional volunteers with strict exclusion criteria, which may have created a relatively “super-healthy” cohort and introduced spectrum bias. Second, apart from age and sex, groups were not matched for all potential confounders, and residual effects of low-grade inflammation, diet, body composition and medications cannot be excluded. Therefore, the reported AUC values may overestimate diagnostic performance, and external prospective studies in more heterogeneous clinical populations are needed to validate these findings.

## Figures and Tables

**Table 1:** Comparison of demographic characteristics and kynurenine pathway metabolites and enzymes in patients with pituitary adenoma and healthy controls.

		Pituitary Adenoma Patient Group (N = 50)	Healthy Control Group (N = 35)	Effect Size (95% CI)	*p* Value
**Gender**	**Female**	41 (63.1)	24 (36.9)		0.151
	**Male**	9 (55)	11 (55)		
**Age (year)**		37 ± 11.2	40.8 ±11.1		0.157
**TSH (mU/L)**		1.42 ± 0.79			NA
**WBC (×10^3^/µL)**		7.49 (6.53–8.7)	-		NA
**Hemoglobin (g/dL)**		13.25 (12.4–14.4)	-		NA
**Platelet (×10^3^/µL)**		289.5 (246–319)	-		NA
**Neutrophil (×10^3^/µL)**		4190 (3530–5170)	-		NA
**Lymphocyte (×10^3^/µL)**		2060 (1750–2700)	-		NA
**Creatinine (mg/dL)**		0.7 (0.6–0.9)	-		NA
**Prolactin (ng/mL)**		11.5 (6–21.16)	-		NA
**ACTH (pmol/L)**		17.25 (9–27.6)	-		NA
**GH (µg/L)**		0.8 (0.44–2.3)	-		NA
**CRP**		2.8 (2–5.1)	-		NA
**ALT (U/L)**		15.1 (11.2–21.1)	-		NA
**AST (U/L)**		20.4 ± 6.61	-		NA
**Total Testosterone**		2.09 (1.2–5)	-		NA
**Tumor diameter (CM)**		1.1 (0.7–1.8)	-		NA
**Tryptophan (µg/mL)**		45.8 ± 10.6	42.7 ± 11.7	0.28 (−0.15–0.71)	0.199
**Kynurenine (ng/mL)**		102 (88.8–112)	78.6 (58.2–89.2)	1.30 (0.87–1.72)	0.001
**Kynurenic Acid (ng/mL)**		122 ± 25.9	82.9 ± 18.7	1.67 (1.17–2.17)	0.001
**IDO (ng/mL)**		14.7 ± 2.89	9.8 ± 3.32	1.58 (1.09–2.07)	0.001
**Kynureninase (ng/mL)**		18.8 ± 4.75	16.8 ± 6.75	0.35 (−0.08–0.78)	0.096
**Kynurenine Aminotransferase (ng/mL)**		14.9 ± 2.79	7.99 ± 3.78	2.12 (1.58–2.65)	0.001
**3-hydroxy kynureninase (ng/mL)**		21.9 ± 5.79	20.8 ± 6.84	0.28 (−0.25–0.6)	0.378
**Anthranilic Acid (ng/mL)**		19.5 (13.5–26.5)	20.9 (17.1–30.2)	−0.3 (−0.73–0.13)	0.151
**Quinolinic acid (mmol/mL)**		1590 ± 811	916 ± 375	1.01 (0.61–1.41)	0.001
**Picolinic Acid (µmol/mL)**		2540 ± 822	1987 ± 756	0.7 (0.3–1.09)	0.001

**Table 2:** Univariate and age/sex-adjusted multivariate binary logistic regression analyses of kynurenine pathway biomarkers associated with pituitary adenoma. OR: odds ratio; CI: confidence interval.

**Univariate Binary Logistic Regression**							
	**B**	**Wald**	***p* Value**	**Odds Raito (Exp(B))**	**95% CI for EXP(B)**		
					**Lower**		**Upper**
**Gender**	−0.736	2.022	0.155	0.479	0.174		1.321
**Age (year)**	−0.028	1.991	0.158	0.972	0.935		1.011
**Quinolinic acid (mmol/mL)**	0.002	14.15	**0.001**	1.002	1.001		1.003
**Picolinic Acid (µmol/mL)**	0.001	8.308	**0.004**	1.001	1.00		1.002
**IDO (ng/mL)**	0.523	20.969	**0.001**	1.688	1.349		2.111
**Kynurenic Acid (ng/mL)**	0.088	22.595	**0.001**	1.091	1.053		1.132
**Kynurenine (ng/mL)**	0.063	17.346	**0.001**	1.065	1.034		1.097
**Tryptophan (µg/mL)**	0.026	1.650	0.199	1.027	0.986		1.069
**Kynureninase (ng/mL)**	0.067	2.721	0.099	1.069	0.987		1.158
**Kynurenine aminotransferase (ng/mL)**	0.595	22.463	**0.001**	1.813	1.413		2.319
**3-hydroxy kynureninase (ng/mL)**	0.032	0.790	0.374	1.032	0.962		1.108
**Anthranilic Acid (ng/mL)**	−0.024	1.535	0.215	0.976	0.94		1.014
**Multivariate Binary Logistic Regression**							
	**B**	**Wald**	***p* Value**	**Odds Raito (Exp(B))**	**95% CI for EXP(B)**		**VIF Score**
					**Lower**	**Upper**	
**Gender**	−0.358	0.036	0.850	0.699	0.017	28.631	1.174
**Age (year)**	−0.097	1.583	0.208	0.908	0.781	1.056	1.119
**IDO (ng/mL)**	1.006	5.997	**0.014**	2.736	1.223	6.122	1.239
**Kynurenic Acid (ng/mL)**	0.144	6.203	0.013	1.155	1.031	1.293	1.226
**Kynurenine (ng/mL)**	0.099	3.308	**0.009**	1.104	0.992	1.229	1.207

Bold values *p* < 0.005 are statistically significant.

**Table 3:** Performance Metrics and Optimal Cutoff Values of Kynurenine Pathway Metabolites Considered Important for the Diagnosis of Pituitary Adenoma.

	Cutoff	AUC (95% CI)	Sensitivity (95% CI)	Specificity (95% CI)
**Kynurenine**	>89.93	0.81 (0.71–0.887)	72 (57–82)	83 (73–92)
**Kynurenic Acid**	>100.59	0.901 (0.816–0.955)	76 (61–86)	89 (73–96)
**IDO**	>12.41	0.874 (0.785–0.936)	82 (68–91)	83 (66–93)
**Kynurenine Aminotransferase**	>10.08	0.923 (0.845–0.970)	96 (86–99)	77 (60–89)
**Quinolinic Acis**	>1305	0.763 (0.659–0.849)	68 (53–80)	91 (76–98)
**Picolinic Acid**	>2176	0.698 (0.589–0.793)	68 (53–80)	69 (50–83)

## Data Availability

The original contributions presented in this study are included in the article; further inquiries can be directed to the corresponding authors.

## Supplementary Materials

The following supporting information can be downloaded at https://www.mdpi.com/article/10.3390/medicina61122120/s1, Table S1: The all correlation analysis; Table S2: Cross-validated performance of PLS-DA models with increasing numbers of components for discrimination between pituitary adenoma patients and healthy controls; Figure S1: The ROC curves for all kynurenine pathway-related variables to discriminate Pituitary Adenoma patients and the Healthy controls; Figure S2: Ten-fold cross-validated receiver operating characteristic (ROC) curve for the multivariable logistic regression model (sex, age, IDO, kynurenic acid and kynurenine) discriminating pituitary adenoma patients from healthy controls. The cross-validated area under the curve (AUC) was 0.963 with a 95% confidence interval of 0.930–0.997.
