# Supplementary material for: Serum Kynurenine Pathway Metabolites as Candidate Diagnostic Biomarkers for Pituitary Adenoma: A Case–Control Study
Source: Medicina (Kaunas). 2025 Nov 28;61(12):2120. doi: 10.3390/medicina61122120 (PMC12734508; doi:10.3390/medicina61122120)
Supplement: Supplementary file 1 [file medicina-61-02120-s001.zip › medicina-3971847-supplementary.pdf]

Table S1: All correlation analyses.

		Age (year)	TSH (mU/L)	WBC (x103/µL)	Hemoglobin (g/dL)	Platelet (x103/µL)	Neutrophil (x103/µL)	Lymphocyte (x103/µL)	Creatinine (mg/dL)	Prolactin (ng/mL)	ACTH (pmol/L)	GH (µg/L)	CRP	ALT (U/L)	AST (U/L)	Total Testosterone	Tumor diameter	Tryptophan (µg/mL)	Kynurenine (ng/mL)	Kynurenic Acid (ng/mL)	IDO (ng/mL)	Kynureninase (ng/mL)	Kynurenine Aminotransferase (ng/mL)	3-hydroxy kynureninase (ng/mL)	Anthranilic Acid (ng/mL)	Quinolinic acid (mmol/mL)	Picolinic Acid (µmol/mL)
Age (year)	Pearson Correlation	1	,247	-,003	,303*	,141	,164	,154	,248	-,250	,009	,012	-,108	,174	,009	-,168	,340*	-,153	,055	-,008	,112	,041	-,061	-,096	,365**	,228	,202
	Sig. (2-tailed)		,084	,984	,032	,330	,256	,285	,083	,079	,953	,936	,455	,227	,950	,245	,016	,288	,704	,958	,441	,778	,676	,508	,009	,111	,159
	N	50	50	50	50	50	50	50	50	50	50	50	50	50	50	50	50	50	50	50	50	50	50	50	50	50	50
TSH (mU/L)	Pearson Correlation	,247	1	-,068	-,132	-,143	-,014	-,036	-,072	-,027	-,255	,143	-,095	,271	,041	-,087	,302*	,037	,231	-,104	-,061	,130	,255	,022	-,050	,006	,072
	Sig. (2-tailed)	,084		,637	,361	,323	,920	,806	,620	,855	,074	,323	,512	,057	,779	,548	,033	,798	,107	,473	,674	,367	,074	,880	,730	,969	,619
	N	50	50	50	50	50	50	50	50	50	50	50	50	50	50	50	50	50	50	50	50	50	50	50	50	50	50
WBC (x103/µL)	Pearson Correlation	-,003	-,068	1	,184	,066	,519**	,380**	,164	-,130	-,201	-,140	-,194	,212	-,008	,246	-,010	,230	-,014	,151	-,158	-,101	,108	,206	,014	,281*	,163
	Sig. (2-tailed)	,984	,637		,201	,650	,000	,006	,254	,369	,161	,332	,177	,139	,957	,085	,943	,108	,925	,296	,272	,484	,454	,151	,922	,048	,260
	N	50	50	50	50	50	50	50	50	50	50	50	50	50	50	50	50	50	50	50	50	50	50	50	50	50	50
Hemoglobin (g/dL)	Pearson Correlation	,303*	-,132	,184	1	-,028	,130	,053	,193	-,032	,204	,018	,142	-,075	,036	,088	,087	-,250	-,043	,241	-,004	-,071	-,154	-,007	,179	,121	-,263
	Sig. (2-tailed)	,032	,361	,201		,845	,367	,716	,180	,827	,156	,904	,324	,606	,806	,545	,549	,080	,765	,092	,976	,623	,286	,962	,213	,401	,065
	N	50	50	50	50	50	50	50	50	50	50	50	50	50	50	50	50	50	50	50	50	50	50	50	50	50	50
Platelet (x103/µL)	Pearson Correlation	,141	-,143	,066	-,028	1	,200	,132	-,102	,020	-,250	-,043	,003	,101	,004	,022	-,045	,170	-,274	-,030	,097	,322*	-,082	-,062	,158	,071	,156
	Sig. (2-tailed)	,330	,323	,650	,845		,164	,360	,480	,893	,080	,768	,982	,487	,979	,882	,759	,237	,054	,839	,503	,022	,570	,668	,273	,624	,280
	N	50	50	50	50	50	50	50	50	50	50	50	50	50	50	50	50	50	50	50	50	50	50	50	50	50	50
Neutrophil (x103/µL)	Pearson Correlation	,164	-,014	,519**	,130	,200	1	,298*	,174	,083	-,192	,106	,033	,132	,217	,081	,039	,154	,103	-,002	,181	,099	,054	,078	,185	,230	,179
	Sig. (2-tailed)	,256	,920	,000	,367	,164		,036	,227	,564	,182	,462	,821	,361	,130	,575	,787	,285	,477	,988	,209	,495	,708	,591	,199	,109	,212
	N	50	50	50	50	50	50	50	50	50	50	50	50	50	50	50	50	50	50	50	50	50	50	50	50	50	50
Lymphocyte (x103/µL)	Pearson Correlation	,154	-,036	,380**	,053	,132	,298*	1	,293*	-,049	-,158	-,206	-,169	,523**	,330*	,095	-,063	,116	-,027	-,028	-,024	-,052	-,055	-,184	-,059	,082	,221
	Sig. (2-tailed)	,285	,806	,006	,716	,360	,036		,039	,738	,274	,151	,239	,000	,019	,510	,665	,423	,854	,848	,867	,718	,705	,202	,683	,570	,122
	N	50	50	50	50	50	50	50	50	50	50	50	50	50	50	50	50	50	50	50	50	50	50	50	50	50	50
Creatinine (mg/dL)	Pearson Correlation	,248	-,072	,164	,193	-,102	,174	,293*	1	,003	,002	,075	,048	,119	-,055	,379**	-,057	,064	,074	,010	-,092	,068	-,065	-,048	,211	-,068	,128
	Sig. (2-tailed)	,083	,620	,254	,180	,480	,227	,039		,985	,989	,607	,743	,410	,702	,007	,695	,659	,610	,945	,525	,637	,653	,739	,141	,640	,375
	N	50	50	50	50	50	50	50	50	50	50	50	50	50	50	50	50	50	50	50	50	50	50	50	50	50	50
Prolactin (ng/mL)	Pearson Correlation	-,250	-,027	-,130	-,032	,020	,083	-,049	,003	1	-,065	,227	,032	-,101	,005	-,073	-,208	-,201	-,101	-,040	-,025	,145	,012	-,082	-,028	-,134	-,123
	Sig. (2-tailed)	,079	,855	,369	,827	,893	,564	,738	,985		,652	,113	,824	,485	,974	,617	,147	,162	,484	,785	,861	,314	,935	,572	,848	,355	,397
	N	50	50	50	50	50	50	50	50	50	50	50	50	50	50	50	50	50	50	50	50	50	50	50	50	50	50
ACTH (pmol/L)	Pearson Correlation	,009	-,255	-,201	,204	-,250	-,192	-,158	,002	-,065	1	-,204	-,087	-,196	-,055	-,150	,023	-,177	-,114	,127	,132	-,225	,006	-,023	,009	,116	-,134
	Sig. (2-tailed)	,953	,074	,161	,156	,080	,182	,274	,989	,652		,154	,548	,172	,702	,299	,873	,218	,431	,380	,360	,116	,965	,873	,953	,424	,354
	N	50	50	50	50	50	50	50	50	50	50	50	50	50	50	50	50	50	50	50	50	50	50	50	50	50	50
GH (µg/L)	Pearson Correlation	,012	,143	-,140	,018	-,043	,106	-,206	,075	,227	-,204	1	,502**	-,119	-,039	-,117	,099	,049	,197	-,040	-,063	,192	-,243	-,056	,185	-,128	,072
	Sig. (2-tailed)	,936	,323	,332	,904	,768	,462	,151	,607	,113	,154		,000	,410	,789	,419	,494	,737	,170	,783	,663	,182	,089	,697	,199	,377	,619
	N	50	50	50	50	50	50	50	50	50	50	50	50	50	50	50	50	50	50	50	50	50	50	50	50	50	50
CRP	Pearson Correlation	-,108	-,095	-,194	,142	,003	,033	-,169	,048	,032	-,087	,502**	1	-,124	,030	-,147	-,026	-,056	,165	-,073	,038	-,076	-,253	-,001	,338*	-,225	-,170
	Sig. (2-tailed)	,455	,512	,177	,324	,982	,821	,239	,743	,824	,548	,000		,390	,837	,309	,860	,701	,253	,616	,792	,599	,076	,996	,017	,116	,237
	N	50	50	50	50	50	50	50	50	50	50	50	50	50	50	50	50	50	50	50	50	50	50	50	50	50	50
ALT (U/L)	Pearson Correlation	,174	,271	,212	-,075	,101	,132	,523**	,119	-,101	-,196	-,119	-,124	1	,313*	,133	,036	,032	-,025	-,040	,242	,005	,092	,083	,075	,039	,071
	Sig. (2-tailed)	,227	,057	,139	,606	,487	,361	,000	,410	,485	,172	,410	,390		,027	,359	,804	,823	,865	,784	,090	,975	,525	,567	,605	,786	,623
	N	50	50	50	50	50	50	50	50	50	50	50	50	50	50	50	50	50	50	50	50	50	50	50	50	50	50
AST (U/L)	Pearson Correlation	,009	,041	-,008	,036	,004	,217	,330*	-,055	,005	-,055	-,039	,030	,313*	1	-,048	-,006	-,006	-,056	-,055	,128	-,149	-,068	-,322*	,047	-,084	,152
	Sig. (2-tailed)	,950	,779	,957	,806	,979	,130	,019	,702	,974	,702	,789	,837	,027		,743	,964	,968	,697	,702	,377	,302	,640	,022	,747	,564	,292
	N	50	50	50	50	50	50	50	50	50	50	50	50	50	50	50	50	50	50	50	50	50	50	50	50	50	50
Total Testosterone	Pearson Correlation	-,168	-,087	,246	,088	,022	,081	,095	,379**	-,073	-,150	-,117	-,147	,133	-,048	1	-,151	,159	,048	,119	-,085	,345*	-,119	,167	-,148	,108	,031
	Sig. (2-tailed)	,245	,548	,085	,545	,882	,575	,510	,007	,617	,299	,419	,309	,359	,743		,294	,271	,739	,410	,556	,014	,411	,246	,304	,457	,829
	N	50	50	50	50	50	50	50	50	50	50	50	50	50	50	50	50	50	50	50	50	50	50	50	50	50	50
Tumor diameter	Pearson Correlation	,340*	,302*	-,010	,087	-,045	,039	-,063	-,057	-,208	,023	,099	-,026	,036	-,006	-,151	1	-,166	,343*	-,121	-,202	-,047	-,068	,034	,214	,195	,144

	Sig. (2-tailed)	,016	,033	,943	,549	,759	,787	,665	,695	,147	,873	,494	,860	,804	,964	,294		,248	,015	,404	,159	,744	,637	,812	,136	,175	,318
	N	50	50	50	50	50	50	50	50	50	50	50	50	50	50	50	50	50	50	50	50	50	50	50	50	50	50
Tryptophan (µg/mL)	Pearson Correlation	-,153	,037	,230	-,250	,170	,154	,116	,064	-,201	-,177	,049	-,056	,032	-,006	,159	-,166	1	,044	,153	,019	,001	-,026	-,012	-,086	-,021	,074
	Sig. (2-tailed)	,288	,798	,108	,080	,237	,285	,423	,659	,162	,218	,737	,701	,823	,968	,271	,248		,759	,289	,897	,992	,860	,932	,551	,887	,608
	N	50	50	50	50	50	50	50	50	50	50	50	50	50	50	50	50	50	50	50	50	50	50	50	50	50	50
Kynurenine (ng/mL)	Pearson Correlation	,055	,231	-,014	-,043	-,274	,103	-,027	,074	-,101	-,114	,197	,165	-,025	-,056	,048	,343*	,044	1	-,094	-,090	,050	-,084	,100	-,133	,005	-,001
	Sig. (2-tailed)	,704	,107	,925	,765	,054	,477	,854	,610	,484	,431	,170	,253	,865	,697	,739	,015	,759		,515	,534	,731	,562	,490	,357	,973	,995
	N	50	50	50	50	50	50	50	50	50	50	50	50	50	50	50	50	50	50	50	50	50	50	50	50	50	50
Kynurenic Acid (ng/mL)	Pearson Correlation	-,008	-,104	,151	,241	-,030	-,002	-,028	,010	-,040	,127	-,040	-,073	-,040	-,055	,119	-,121	,153	-,094	1	-,103	-,187	,095	,178	-,102	-,013	,017
	Sig. (2-tailed)	,958	,473	,296	,092	,839	,988	,848	,945	,785	,380	,783	,616	,784	,702	,410	,404	,289	,515		,477	,193	,512	,217	,481	,928	,907
	N	50	50	50	50	50	50	50	50	50	50	50	50	50	50	50	50	50	50	50	50	50	50	50	50	50	50
IDO (ng/mL)	Pearson Correlation	,112	-,061	-,158	-,004	,097	,181	-,024	-,092	-,025	,132	-,063	,038	,242	,128	-,085	-,202	,019	-,090	-,103	1	,111	,070	,086	-,075	,109	,023
	Sig. (2-tailed)	,441	,674	,272	,976	,503	,209	,867	,525	,861	,360	,663	,792	,090	,377	,556	,159	,897	,534	,477		,443	,627	,552	,606	,452	,872
	N	50	50	50	50	50	50	50	50	50	50	50	50	50	50	50	50	50	50	50	50	50	50	50	50	50	50
Kynureninase (ng/mL)	Pearson Correlation	,041	,130	-,101	-,071	,322*	,099	-,052	,068	,145	-,225	,192	-,076	,005	-,149	,345*	-,047	,001	,050	-,187	,111	1	-,100	-,094	-,001	,030	,025
	Sig. (2-tailed)	,778	,367	,484	,623	,022	,495	,718	,637	,314	,116	,182	,599	,975	,302	,014	,744	,992	,731	,193	,443		,488	,516	,997	,837	,864
	N	50	50	50	50	50	50	50	50	50	50	50	50	50	50	50	50	50	50	50	50	50	50	50	50	50	50
Kynurenine Aminotransferase (ng/mL)	Pearson Correlation	-,061	,255	,108	-,154	-,082	,054	-,055	-,065	,012	,006	-,243	-,253	,092	-,068	-,119	-,068	-,026	-,084	,095	,070	-,100	1	,053	-,167	,112	-,003
	Sig. (2-tailed)	,676	,074	,454	,286	,570	,708	,705	,653	,935	,965	,089	,076	,525	,640	,411	,637	,860	,562	,512	,627	,488		,716	,245	,438	,985
	N	50	50	50	50	50	50	50	50	50	50	50	50	50	50	50	50	50	50	50	50	50	50	50	50	50	50
3-hydroxy kynureninase (ng/mL)	Pearson Correlation	-,096	,022	,206	-,007	-,062	,078	-,184	-,048	-,082	-,023	-,056	-,001	,083	-,322*	,167	,034	-,012	,100	,178	,086	-,094	,053	1	-,250	,019	,067
	Sig. (2-tailed)	,508	,880	,151	,962	,668	,591	,202	,739	,572	,873	,697	,996	,567	,022	,246	,812	,932	,490	,217	,552	,516	,716		,080	,898	,645
	N	50	50	50	50	50	50	50	50	50	50	50	50	50	50	50	50	50	50	50	50	50	50	50	50	50	50
Anthranilic Acid (ng/mL)	Pearson Correlation	,365**	-,050	,014	,179	,158	,185	-,059	,211	-,028	,009	,185	,338*	,075	,047	-,148	,214	-,086	-,133	-,102	-,075	-,001	-,167	-,250	1	-,070	-,100
	Sig. (2-tailed)	,009	,730	,922	,213	,273	,199	,683	,141	,848	,953	,199	,017	,605	,747	,304	,136	,551	,357	,481	,606	,997	,245	,080		,630	,490
	N	50	50	50	50	50	50	50	50	50	50	50	50	50	50	50	50	50	50	50	50	50	50	50	50	50	50
Quinolinic acid (mmol/mL)	Pearson Correlation	,228	,006	,281*	,121	,071	,230	,082	-,068	-,134	,116	-,128	-,225	,039	-,084	,108	,195	-,021	,005	-,013	,109	,030	,112	,019	-,070	1	,071
	Sig. (2-tailed)	,111	,969	,048	,401	,624	,109	,570	,640	,355	,424	,377	,116	,786	,564	,457	,175	,887	,973	,928	,452	,837	,438	,898	,630		,626
	N	50	50	50	50	50	50	50	50	50	50	50	50	50	50	50	50	50	50	50	50	50	50	50	50	50	50
Picolinic Acid (µmol/mL)	Pearson Correlation	,202	,072	,163	-,263	,156	,179	,221	,128	-,123	-,134	,072	-,170	,071	,152	,031	,144	,074	-,001	,017	,023	,025	-,003	,067	-,100	,071	1
	Sig. (2-tailed)	,159	,619	,260	,065	,280	,212	,122	,375	,397	,354	,619	,237	,623	,292	,829	,318	,608	,995	,907	,872	,864	,985	,645	,490	,626	
	N	50	50	50	50	50	50	50	50	50	50	50	50	50	50	50	50	50	50	50	50	50	50	50	50	50	50

Table S2: Cross-validated performance of PLS-DA models with increasing numbers of components for discrimination between pituitary adenoma patients and healthy controls.

**Number of components**	**Accuracy (10-fold CV)**	R^2	Q^2
1 comps	0.69583	0.20798	0.15696
2 comps	0.6666	0.22025	0.16498

**Number of components**	**Accuracy (10-fold CV)**	**R^2**	**Q^2**
3 comps	0.8	0.41552	0.30349
4 comps	0.7875	0.44287	0.30171
5 comps	0.78472	0.54071	0.22396
6 comps	0.80694	0.5583	0.33326
7 comps	0.80694	0.56511	0.3472
8 comps	0.81944	0.56649	0.34226

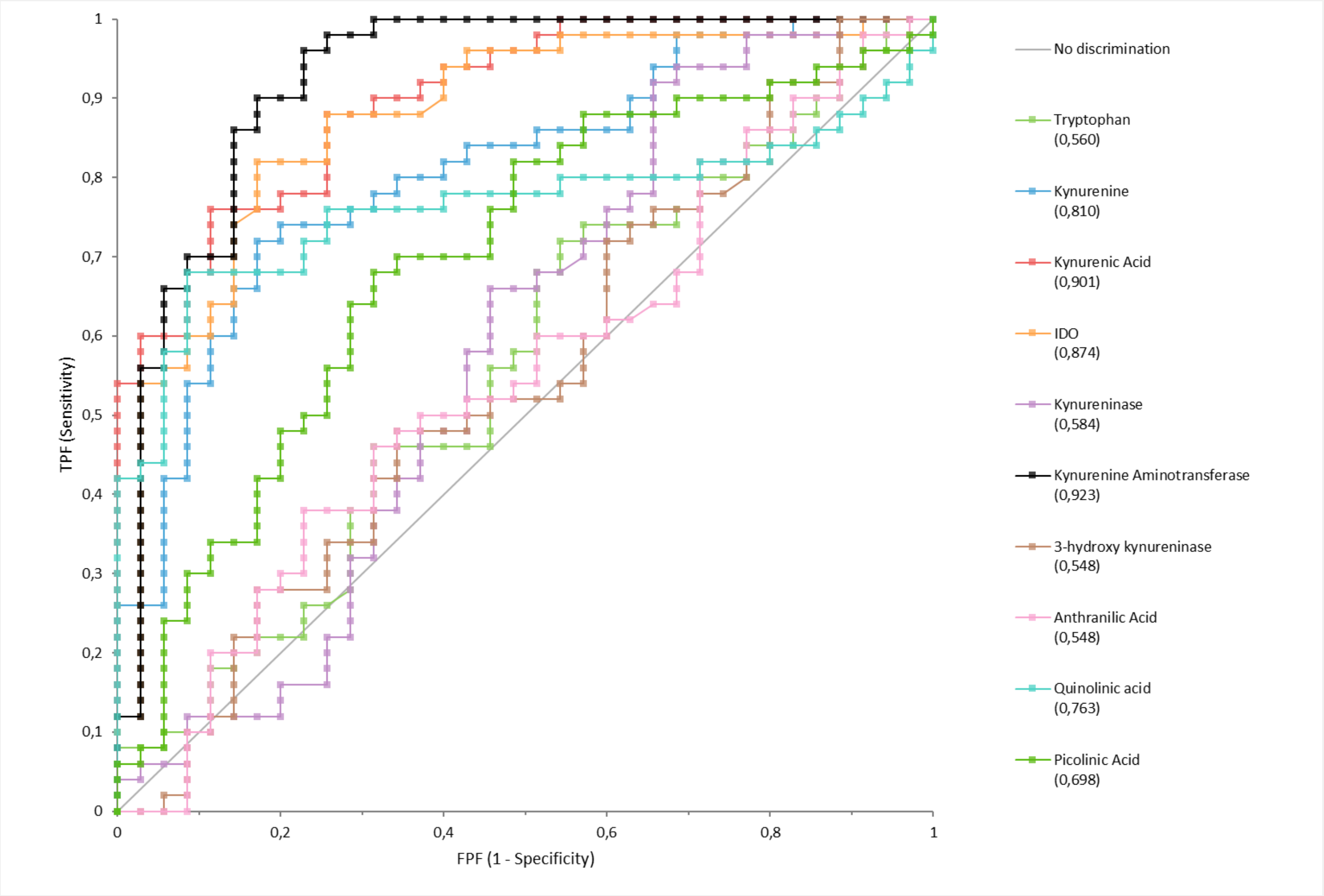

Figure S1: The ROC curves for all kynurenine pathway-related variables to discriminate pituitary adenoma patients and the healthy controls.

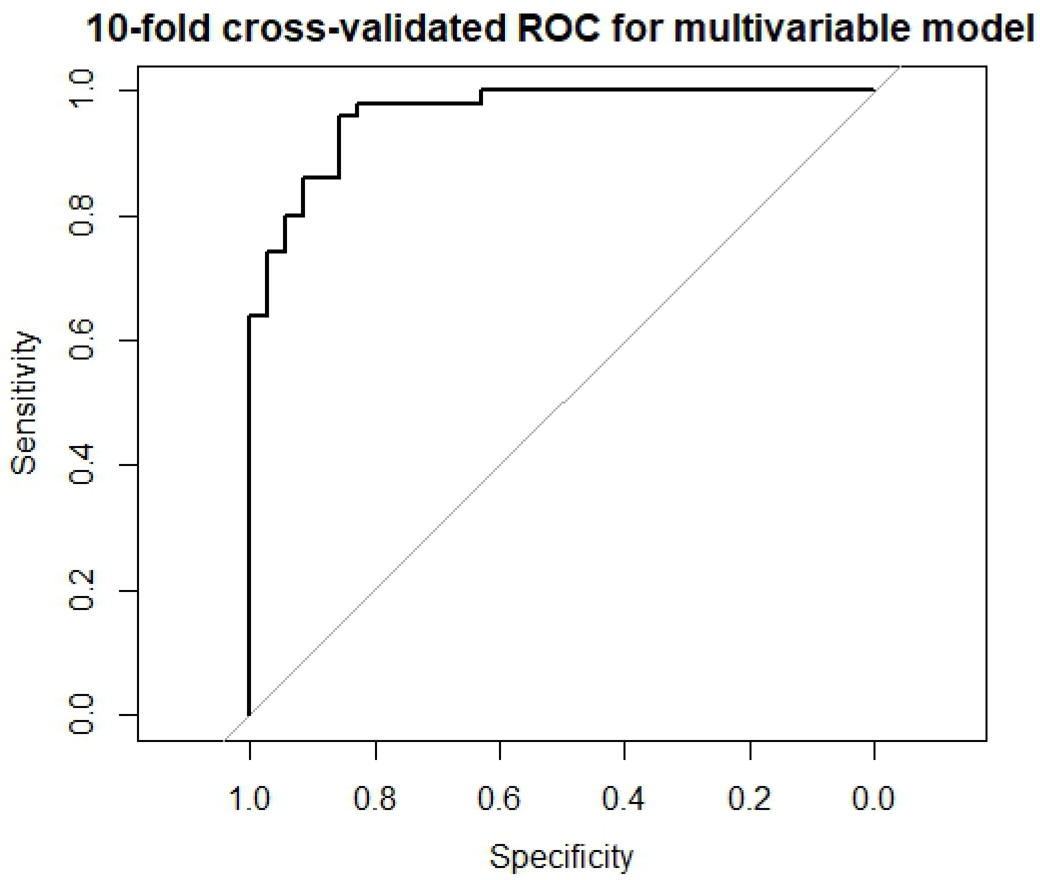

Figure S2: Ten-fold cross-validated receiver operating characteristic (ROC) curve for the multivariable logistic regression model (sex, age, IDO, kynurenic acid and kynurenine) discriminating pituitary adenoma patients from healthy controls. The cross-validated area under the curve (AUC) was 0.963 with a 95% confidence interval of 0.930–0.997.
